# Effect of αlipoic acid and silymarin on bladder outlet obstruction

**DOI:** 10.3892/etm.2012.831

**Published:** 2012-11-23

**Authors:** ABIDIN YILDIRIM, BARBAROS BAŞESKIOĞLU, HALIDE E. TEMEL, NILÜFER ERKASAP, AYDIN YENILMEZ, SEMA USLU, CANER ÖZER, METE OZKURT, TURGUT DÖNMEZ

**Affiliations:** 1 Department of Urology, Faculty of Medicine, Osmangazi University; Eskişehir, Turkey; 2 Department of Biochemistry, Faculty of Pharmacy, Anadolu University; Eskişehir, Turkey; 3 Department of Physiology, Faculty of Medicine, Osmangazi University, Eskişehir, Turkey; 4 Department of Biochemistry, Faculty of Medicine, Osmangazi University, Eskişehir, Turkey; 5 Department of Histology, Faculty of Medicine, Osmangazi University, Eskişehir, Turkey

**Keywords:** urinary bladder, bladder outlet obstruction, αlipoic acid, silymarin, rat

## Abstract

The aim of the present study was to determine whether the treatment of obstructed rat bladders with αlipoic acid (ALA) and silymarin reverses the biochemical and physiological responses to bladder outlet obstruction (BOO). A total of 32 adult Sprague Dawley rats were divided into four groups (n=8 per group): sham (placebo surgery) animals with no treatment (group 1); control animals with surgically induced BOO (group 2); obstructed rats treated with ALA (group 3); and obstructed rats treated with silymarin (group 4). Histological evaluation, bladder weights, collagen structure, TdT-mediated biotin nick end-labeling (TUNEL), inducible nitric oxide sentase (iNOS) mRNA levels, malondialdehyde (MDA) levels and tumor necrosis factor (TNF) levels were investigated. The ALA-treated group had similar bladder weights, collagen levels and TUNEL positivity and decreased iNOS levels compared with the control group, while the silymarin group exhibited further differences. Serum MDA and TNF-α levels were both decreased in the ALA and silymarin groups. ALA treatment reduced the increased oxidative stress and bladder inflammation caused by BOO and may contribute to the protection of bladder function.

## Introduction

Bladder outlet obstruction (BOO) developing secondary to benign prostatic hyperplasia (BPH) is observed at varying degrees in ∼80% of male patients over the age of 50 years (1). Increased wall thickness induced by BOO augments the bladder injury by resulting in cyclic ischemia/reperfusion (I/R) injury during each urination (2). I/R injury in the bladder leads to the generation of reactive oxygen species which may be a pathogenic factor in the inflammation of the dysfunctional detrusor and thus antioxidants may be beneficial for treating bladder dysfunction secondary to BPH/BOO (3). Major cytokines stimulating apoptosis include tumor necrosis factor (TNF)-α, interleukin (IL)-6 and inducible nitric oxide synthase (iNOS) which have been observed to be present at increased levels in the inflammation of rat bladder (4,5).

Similarly, the permanence of membrane injury may also prepare the ground for partial BOO-associated progressive bladder dysfunction (2). Increased bladder outlet resistance results in decreased compliance and increased pressure, as well as a loss of bladder viscoelasticity due to fibroproliferative development in the mucosa and collagen accumulation. Collagen production is of critical significance in this process. Although type III collagen is elastic in character, collagen that is first produced by fibroblasts and then by muscle cells destroys the viscoelastic character of the bladder, leading to poor compliance (6).

Studies have been conducted on the efficacy of αlipoic acid (ALA), an ideal, unique and universal antioxidant, on bladder contractility in animal models of partial BOO. However, to the best of our knowledge, no studies had been performed on the effects of ALA on type I and III collagen ratios and iNOS mRNA gene expression, as well as on cytokines such as TNF-α and IL-6 which are involved in apoptosis and oxidative injury in the bladder. It was proposed that these parameters should be investigated to observe the I/R injury occurring in BOO and to evaluate the efficacy of antioxidant therapy. Silymarin has anticarcinogenic, antiapoptotic and antioxidant characteristics. It also favors cell proliferation, while offering protection against neurotoxins and cardiotoxins, with estrogenic and antiestrogenic effects (7,8). Despite the protective character of silymarin in various tissues and organs, no studies have been conducted with regard to its effects on the bladder to the best of our knowledge.

We proposed that the use of antioxidants may avoid the release of free oxygen radicals during reperfusion. The decision was made to use ALA and silymarin as antioxidant agents in the present study to provide protection against reperfusion injury, the underlying cause of bladder injury.

## Materials and methods

### Animals

A total of 32 6-month-old female Sprague Dawley rats weighing 200–250 g were used in the study. Approval was obtained (dated 13.05.2009, no. 19/111) from the ESOGÜ Local Ethics Committee on Animal Experiments for all procedures that were to be performed on animals.

The rats were divided into four groups, each consisting of eight rats: group I (sham), group II (BOO), group II (control), group III (ALA, 100mg/kg, intraperitoneally) and group IV (silymarin, 25mg/kg, intraperitoneally). ALA and silymarin were dissolved in dimethyl sulfoxide. The BOO model was same as that of Hashimoto *et al*(9). In the sham group, placebo surgery with a median laparotomy was performed. For the control group, a urethral catheter (1.2 mm) was inserted into the bladder. Following the median laparotomy the bladder and urethra were exposed. A 3-0 suture was placed in the urethrovesical junction. Experimental BOO was completed by removing the urethral catheter. The same procedure was performed for groups III and IV. Medications were then administered intraperitoneally (IP) for one month.

### Experimental protocol

Once the experimental protocol had been completed, the rats were administered general anesthesia with ketamine (75 mg/kg, IP) and xylazine (15 mg/kg, IP) following 12 h of fasting. After reaching the bladder through a suprapubic incision with a median laparotomy, the bladder tissue was resected proximal to the sutured area from the ureterovesical junction. Intracardiac blood samples were collected using a syringe. The rats were then sacrificed by drawing excessive amounts of blood. The bladder weight of each rat was recorded and the bladder was dissected into three equal sections, from the bladder dome to bladder neck. One of the sections was set aside for the histological investigation of the changes in I/R, apoptosis and collagen-detrusor muscle ratio parameters. The samples collected at this stage were evaluated using various techniques such as direct microscopy, immune staining and TdT-mediated biotin nick end-labeling (TUNEL) staining. Blood samples and one-third of the bladder tissue were set aside for investigating the biochemical parameters. I/R parameters, including malondialdehyde (MDA), IL-6 and TNF-α levels, were measured in the bladder tissue and the remaining one-third of the bladder tissue was evaluated in terms of iNOS gene expression using real-time PCR (RT-PCR).

### Histological evaluation

The tissue samples obtained were immediately immersion fixed in neutral-buffered formalin for 24 h prior to processing and embedding in paraffin wax. Sections (5 μm) were cut using a microtome and stained with Masson's trichrome to examine the smooth muscle/collagen ratio. Slides were examined using an Olympus BX51 light microscope and photographed with an Olympus DP70 camera. The slides were analyzed using a ocular micrometer with a BAB Bs 200 ProP image analysis system.

### Immunohistochemical evaluation of collogen types I and III

The primary antibodies were rabbit polyclonal collagen type I (Abcam, Cambridge, MA, USA; ab292) vs. mouse monoclonal collagen type III (Abcam; ab6310). The collagen type I and collagen type III analysis was performed by two examiners using the modified scoring system introduced by Cör *et al*(10). A minimal staining reaction was scored as (+), a medium staining reaction as (++) and a markedly positive staining reaction as (+++). The data were analyzed and compared in relation to the collagen type I and collagen type III content in both groups.

### TUNEL staining

The DNA fragmentation characteristic of apoptosis was detected with a TUNEL assay and using an Apoptag Plus Peroxidase In Situ Apoptosis Detection kit (S7101, Chemicon International, Temecula, CA, USA). TUNEL evaluation was performed by two blinded investigators. The evaluations were performed on 25 randomly selected sections at x40 magnification in at least 20 areas. TUNEL (+) cells were counted to establish the apoptotic index. Apoptotic cells in the bladder sections of the experimental groups were regarded as TUNEL (+) cells.

### Bladder tissue and serum MDA levels

Lipid peroxidation was assayed by measuring MDA levels on the basis of MDA reacted with thiobarbituric acid (TBA) in tissue homogenates and serum samples, according to Ohkawa *et al*(11). Serum and tissue MDA levels were expressed in nmol/ml and nmol/mg protein, respectively. Tissue protein levels were determined according to the Biuret method.

IL-6 and TNF-α concentrations were measured using enzyme-linked immunosorbent assays (Bender MedSystems, GmbH, Vienna, Austria) according to the manufacturer's instructions using a Victor/X3 microplate reader (Perkin Elmer, Waltham, MA, USA).

### RNA extraction and RT-PCR

The mRNA level of iNOS relative to the housekeeping gene glyceraldehyde 3-phosphate dehydrogenase (GAPDH) was determined using RT-PCR with a Taqman probe. Total RNA was extracted from the renal tissue using the RNA stabilization reagent (Qiagen, Hilden, Germany), according to the manufacturer's instructions and quantified by measuring the absorbance at 260 nm (Nanodrop1000; Thermo, Wilmington, DE, USA). Aliquots (20 μl) of RNA from each group were used for the production of comple mentary DNA (cDNA). The newly synthesized cDNA, stored at −20°C, was used for the mRNA assay of the iNOS isoform with RT-PCR. cDNA (5 μl) from each group was amplified in 20 μl reaction mixture. RT-PCR was performed by monitoring in real time the increase in the amount of Taqman probe using a Rotor-Gene 6000 RT-PCR system (Qiagen). The oligonucleotide sequences of the cDNA primers were designed at Gene Research Laboratories UK (Southampton, UK). The forward primer for rat iNOS was 5′-CACCACCCTCCTTGTTCAAC-3′ and the reverse primer was 5′-CAATCCACAACTCGCTCCAA-3′. Sobajima *et al* also used GAPDH (housekeeping gene) to normalize iNOS (target gene) data using RT-PCR (12).

The RT-PCR thermal cycling conditions were as follows: 15 min at 42°C and 10 min at 4°C for cDNA synthesis, 10 min at 95°C and 20 sec at 95°C, 30 sec at 55°C and 20 sec at 72°C for 50 cycles. RT-PCR data were collected using a Rotor-Gene 6000 detec tion system. Cycle threshold (CT) values were determined using automated threshold analysis. Primer quality (lack of primer-dimer amplification) was confirmed using a melting curve. Relative quantification of the gene expression was performed using the standard curve method, constructed with serial dilutions of control mRNA or RT-PCR amplicons. All experiments were performed in triplicate. iNOS levels were standardized with GAPDH (ratio, iNOS:GAPDH) to account for loading differences. Gene expression levels (mRNA) were reported using the median as a point estimator and the range of values.

### Statistical analysis

SPSS 17.0 and SigmaStat 3.1 software were used for the analyses of all data in the study. Continuous quantitative data were expressed as number, mean and standard deviation, while qualitative data were expressed as numbers and percentages. Continuous data with normal distributions obtained from independent measurements were analyzed using one-way ANOVA, while data with non-normal distributions obtained from independent measurements were analyzed with the Kruskal-Wallis test. Correlation analysis was used to establish the correlation between variables. P<0.05 was considered to indicate statistically significant differences.

## Results

### Histological results

**Figure 1.**
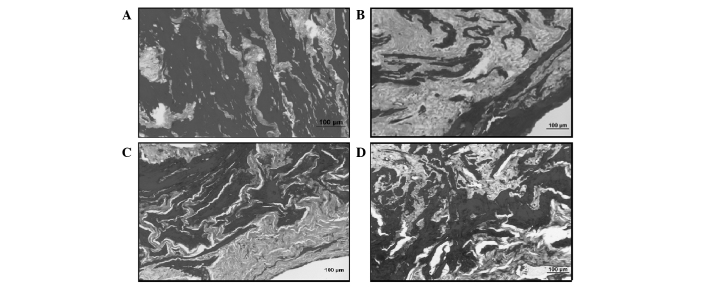
Histological evaluation of smooth muscle bundles. (A) Sham group, smooth muscles were clearly visible. (B) Control group, collagen bundles replaced smooth muscles. (C) Silymarin group, smooth muscles were more protected than in the control group but collagen bundles were also observed. (D) ALA group, small amounts of collagen bundles with more protected smooth muscles. Masson trichrome staining; Bar, 100 *μ*m; ALA, αlipoic acid..

Bladder sections of the control group revealed a multi-layered transitional epithelium and lamina propria with a loose connective tissue layer underneath forming the tunica mucosa. In the tunica muscularis layer, smooth muscle bundles were interrupted by connective tissue fibers. It was noted that the percentages of smooth muscle bundles (stained black) and collagen fibers (stained grey) were equal in the sections stained with Masson's trichrome stain (Fig. 1A). A thicker bladder wall was observed in the sections of the BOO-induced sham group, when compared with the controls. Mild edema, separations and irregularities in the epithelium were noted. The increase in connective tissue among the smooth muscle bundles and in all layers was notable. However, there was a decrease in the smooth muscle quantity. Furthermore, fibroblasts were observed to have increased as well (Fig. 1B). The results observed in the BOO + silymarin group were similar to those noted in the control group. Dense collagen fibers were observed among the smooth muscle bundles (Fig. 1C). By contrast, the BOO + ALA group had relatively equal percentages of collagen and smooth muscle bundles, similar to the sham group. However, edema and irregularities were noted in the epithelium (Fig. 1D).

### Immunohistochemical examination of types I and III collagen

**Figure 2.**
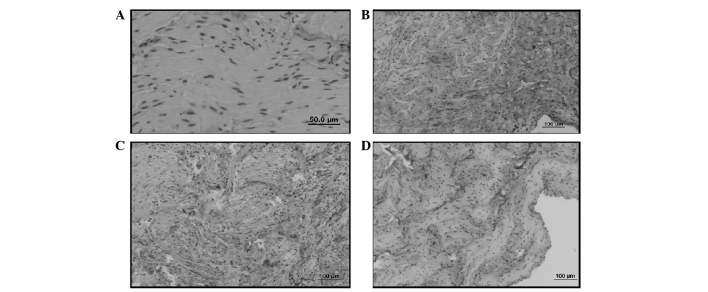
Histological evaluation of collagen type III bundles. (A) Sham group, (B) control group. An increase in the number of type III collagen bundles compared with the sham group and type I collagen was observed. (C) Silymarin group. An increase was observed in collagen type III bundles but there was a slight decrease compared with the control group. (D) ALA group, a decrease in collagen type III bundles compared with the control group was observed. Masson trichrome staining; Bar, 100 *μ*m; ALA, αlipoic acid.

**Figure 3.**
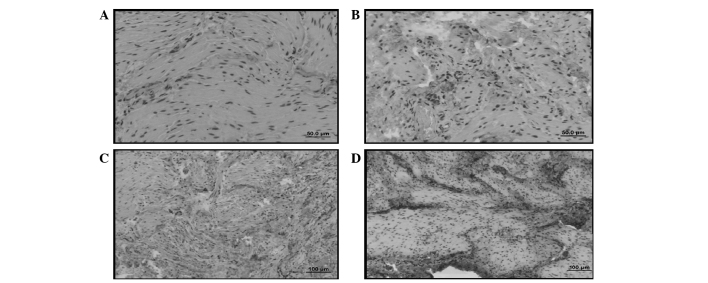
Histological evaluation of collagen I bundles. (A) Sham group, (B) control group. Collagen type I bundles were increased. (C) Silymarin group, (D) ALA group. ALA and silymarin had a similar decreasing effect on collagen type I bundles although higher levels of bundles were observed than in the sham group. Masson trichrome staining; Bar, 100 *μ*m; ALA, αlipoic acid.

In the sham group, type I and type III collagen staining was noted to be generally localized in the lamina propria layer (Figs. 2A and 3A).

In the control group, type III collagen staining was observed to be more marked than in the sham group. Type III collagen staining was also more marked than type I collagen staining in the sham group (Figs. 2B and 3B).

The type III collagen distribution in the BOO + silymarin group was similar to that observed in the control group (Figs. 2C and 3C). Silymarin was observed to be ineffective at reducing the type III collagen levels.

BOO + ALA group was observed as having less intense type I and type III collagen staining when compared with the control group. The type III collagen staining was notably less intense (Figs. 2D and 3D). Histological examination revealed that ALA prevented I/R injury in BOO and reduced the amount of type III collagen, maintaining bladder functions.

### Evaluation of bladder weight

It was observed that the increase in bladder weight in the control group was significantly higher compared with the sham (P<0.001). The bladder weight was similar to that of the sham group in the ALA and silymarin groups and the differences with the control group were statistically significant (P<0.001; Table I).

### TUNEL staining

**Figure 4.**
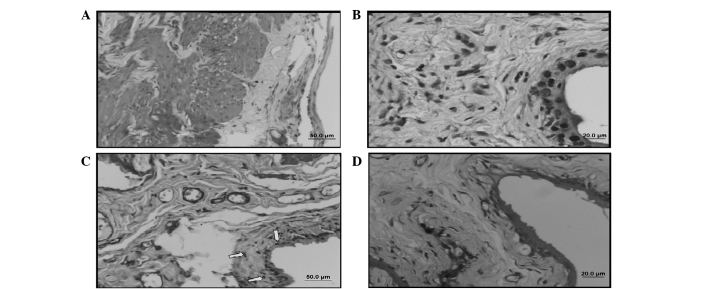
(A) Sham group, (B) control group, an increase in positive cells was observed. (C) Silymarin group, positivity ranged between that of the sham and control. (D) ALA group, significant decrease in TUNEL positivity. Bar, 50 *μ*m; ALA, αlipoic acid; TUNEL, Tdt-mediated biotin nick end-labeling.

TUNEL was evaluated blindly by two examiners. In the sham group, a limited number of TUNEL (+) cells was observed in the epithelium and connective tissue. However, a large number of TUNEL (+) cells was observed in the smooth muscle and epithelium in the control group. The number of TUNEL (+) cells in the BOO + silymarin group was similar in terms of quantity and localization to that of the control group. By contrast, a low number of TUNEL (+) cells was observed in the BOO + ALA group similar to that of the sham group. The number of TUNEL (+) cells in the control group was higher than that observed in the sham group and this increase was statistically significant (P<0.001). Although there was a decrease in the number of TUNEL (+) cells in the ALA- and silymarin-treated groups, the decrease in the silymarin group was not statistically significant. However, the decrease in the number of TUNEL (+) cells was statistically significant in the ALA group (P<0.001). ALA was revealed to be effective at decreasing apoptosis (Table II, Fig. 4).

### Expression of iNOS mRNA in the urinary bladder

Due to the abnormal distribution of the parameters, the Kruskal Wallis test was used and a significant difference in the gene expression levels of iNOS was observed between the groups (P<0.001). To identify the significance of the differences between the groups, the Tukey HSD test was used and iNOS levels in the ALA group were observed to be slightly decreased compared with the control group (P<0.001). However, in the silymarin group this decrease was not significant (P>0.05; Table I).

### Tissue and serum MDA and TNF-α levels

In the ALA and silymarin groups, the decreases in the MDA and TNF-α levels were significant compared with the control group (P<0.001 and P<0.01, respectively). Serum TNF-α levels were observed to be significantly decreased in the ALA and silymarin groups (P<0.05; Table II).

## Discussion

The most significant factor that initiates I/R-associated tissue injury is the presence of free oxygen radicals. The destructive effects of radicals cause cell death as a result of apoptosis and necrosis (3). Similarly, ongoing membrane injury may also be the basis of progressive bladder dysfunction associated with BOO (2). Therefore, while designing the present study the use of antioxidant agents was preferred to protect against free oxygen radicals that are produced during reperfusion and are the main cause of bladder injury.

The increase in bladder wall thickness leads to cyclic I/R injury during each urination and uninhibited contraction. The decrease in blood flow and tissue oxygenation becomes increasingly larger during urination and uninhibited continuous contractions (13). In the present study, a macroscopic increase in the bladder volume was observed in the control group when compared with the sham group following BOO. Studies demonstrated that certain therapeutic agents maintained only the contractile functions of the bladder but offered no protection against the increase in bladder wall thickness and weight. According to current data, the contractile functions of the bladder are maintained in a limited manner prior to the decompensating phase, but as the increase in bladder wall thickness prolongs the I/R injury, the bladder enters into the decompensating stage. ALA and silymarin were observed to be efficacious in terms of providing protection against I/R-induced increases in bladder wall thickness and weight in BOO.

Studies conducted so far have reported no data with regard to the role of TNF-α in bladder I/R injury, to the best of our knowledge. TNF-α released from leukocytes during I/R injury may be a critical parameter for evaluating the injury and assessing the treatment efficacy.

Silymarin has protective properties against hepatic and biliary conditions, with anticarcinogenic, antiapoptotic and antioxidant effects. It promotes cell proliferation and has neuroprotective, cardioprotective, estrogenic and antiestrogenic effects (14,15). Juan *et al* conducted a study in 2008 with rabbits and reported increased bladder weights after four and seven weeks of BOO. The authors demonstrated that the groups undergoing αlipoic acid and coenzyme Q10 treatment had significantly reduced bladder weights when compared with those of the 7-week obstruction group (16).

Saito and Miyagawa measured MDA levels to evaluate lipid peroxidation in I/R injury resulting from acute urinary retention in rats and observed elevated MDA levels following reperfusion and demonstrated that free radicals released during reperfusion led to bladder dysfunction (17). The present study revealed that ALA and silymarin prevented lipid peroxidation and therefore, cell injury resulting from I/R in BOO.

The decrease in the levels of proinflammatory mediators responsible for organ injury, as well as in MDA which is the main cause of oxidative injury, indicated that ALA was effective at providing protection against I/R injury (18).

Several studies investigated TNF-α levels as an indicator of I/R injury in a number of organs, including the kidneys, small intestine, stomach and liver (19–21). However, no studies had been performed to investigate TNF-α and IL-6 levels in I/R injury of the bladder to the best of our knowledge

Elevated levels of TNF-α were observed in the bladder tissue and serum of rats in the control group as a result of I/R injury resulting from BOO, compared with those of the sham group. The results of the present study were consistent with the results reported in studies performed on I/R injury in various organs. The present study demonstrated that antioxidants protect the bladder by inhibiting the release of proinflammatory mediators.

In the present study, it was observed that although ALA had an anti-apoptotic effect in the bladder, silymarin did not. An increase in the number of TUNEL (+) cells in the control group was revealed when compared with the sham group and the increase was statistically significant (P<0.001). The number of TUNEL (+) cells was observed to decrease in the ALA- and silymarin-treated groups, although the decrease in the silymarin group was not significant. By contrast, the decrease in the number of TUNEL (+) cells in the ALA group was significant (P<0.001), indicating the antiapoptotic properties of ALA.

Data obtained previously has revealed that ischemic damage is involved with iNOS, while most iNOS is associated with inflammatory causes (22). However, the role of NO in BOO remains controversial. Several studies have suggested that NO induces cellular cytotoxicity and tissue injury via lipid peroxidation, as well as DNA damage and pro-apoptotic effects in I/R injury (23–25). However, there are studies which demonstrate that the increased activity of NOS is associated with reduced BOO induced injury (26). According to Bae *et al*, the protective effect of ALA treatment in the kidney following I/R was dependent on the reduction of iNOS levels (27). In the present study, quantitative iNOS expression increased in the I/R group and no significant difference was observed between the I/R and silymarin treatment groups, while ALA had significant decreasing effect.

In conclusion, ALA not only has antioxidant and anti-apoptotic activities but also an inhibitory effect on iNOS, collagen formation, lipid peroxidation and TNF-α levels in bladder tissue protection during I/R injury in rats. Therefore, ALA may be used to protect bladder tissue in I/R injury which develops following BOO or during surgical procedures.

## Figures and Tables

**Table I.** Comparison of experimental groups according to bladder weights, TUNEL and iNOS levels.

Variable	Sham	Control	Silymarin	ALA
Bladder weights (g)	0.13±0.05	0.78±0.32^c^	0.32±0.19^f^	0.15±0.05^f^
TUNEL	15.87±4.79	33.12±23.02^c^	22.37±15.30	5.37± 2.92^f^
iNOS mRNA levels	0.117±0.02	6.95±2.43^c^	0.676±0.29	0.246±0.223^f^

a–c Groups vs. sham,

a P<0.05,

b P<0.01,

c P<0.001;

d–f groups vs. control,

d P<0.05,

e P<0.01,

f P<0.001; ALA, αlipoic acid; TUNEL, TdT-mediated biotin nick end-labeling; iNOS, inducible nitric oxide synthase.

**Table II.** Comparison of experimental groups according to serum and tissue levels of MDA and TNF.

Variable	Sham	Control	Silymarin	ALA
Serum MDA	3.1±0.59	4.08±0.89^a^	1.96±0.47^f^	1.36±0.77^f^
Tissue MDA	5.57±1.63	7.53±1.00^a^	2.60±1.22^f^	3.21±1.21^f^
Serum TNF-α	7.17±5.50	14.17±6.88^b^	7.05±3.31^e^	5.31±2.39^e^
Tissue TNF-α	80.27±40.23	127.62±44.80^a^	74.02±19.69^a^	62.31±37.41^a^

a–c Groups vs sham,

a P<0.05,

b P<0.01,

c P<0.001;

d–f groups vs. control,

d P<0.05,

e P<0.01,

f P<0.001; ALA, αlipoic acid. MDA, malondialdehyde; TNF, tumor necrosis factor.

## Abbreviations:

BOO: bladder outlet obstruction
ALA: αlipoic acid
MDA: malondialdehyde
PCR: polymerase chain reaction
iNOS: inducible nitric oxid synthase
I/R: ischemia-reperfusion

## References

[1] Zderic SA, Levin RM, Wein AJ, Gillenwater JY, Grayhack JT, Howards SS, Duckett JW (1996). Voiding function and dysfunction: a relevant anatomy, physiology, and pharmacology, and molecular biology. Adult and Pediatric Urology.

[2] Parekh MH, Lobel R, O'Connor LJ, Leggett RE, Levin RM (2001). Protective effect of vitamin E on the response of the rabbit bladder to partial outlet obstruction. J Urol.

[3] Sahna E, Deniz E, Aksulu HE (2006). Myocardial ischemia-reper-fusion injury and melatonin. Anadolu Kardiyol Derg.

[4] Bonvissuto G, Minutoli L, Morgia G, Bitto A, Polito F, Irrera N, Marini H, Squadrito F, Altavilla D (2011). Effect of Serenoa repens, lycopene, and selenium on proinflammatory phenotype activation: an in vitro and in vivo comparison study. Urology.

[5] Chen LM, Wang C, Chen M, Marcello MR, Chao J, Chao L, Chai KX (2006). Prostasin attenuates inducible nitric oxide synthase expression in lipopolysaccharide-induced urinary bladder inflammation. Am J Physiol Renal Physiol.

[6] Sjuve R, Haase H, Morano, Uvelius B, Arner A (1996). Contraction kinetics and myosin isoform composition in smooth muscle from hypertrophied rat urinary bladder. J Cell Biochem.

[7] Moini H, Packer L, Saris NE (2002). Antioxidant and prooxidant activities of alpha-lipoic acid and dihydrolipoic acid. Toxicology and Applied Pharmacology.

[8] Packer L, Tritschler HJ (1996). Alpha-lipoic acid: the metabolic antioxidant. Free Radic Biol Med.

[9] Hashimoto T, Nagabukuro H, Doi T (2005). Effects of the selective acetylcholinesterase inhibitor TAK-802 on the voiding behavior and bladder mass increase in rats with partial bladder outlet obstruction. J Urol.

[10] Cör A, Barbic M, Kralj B (2003). Differences in the quantity of elastic fibres and collagen type I and type III in endopelvic fascia between women with stress urinary incontinence and controls. Urol Res.

[11] Ohkawa H, Ohishi N, Yagi K (1979). Assay for lipid peroxides in animal tissues by thiobarbituric acid reaction. Anal Biochem.

[12] Sobajima S, Shimer AL, Chadderdon RC, Kompel JF, Kim JS, Gilbertson LG, Kang JD (2005). Quantitative analysis of gene expression in a rabbit model of intervertebral disc degeneration by real-time polymerase chain reaction. Spine J.

[13] Greenland JE, Brading AF (1996). Urinary bladder blood flow changes during the micturition cycle in a conscious pig model. J Urol.

[14] Fraschini F, Demartini G, Esposti D (2002). Pharmacology of Silymarin. Clin Drug Investig.

[15] Kren V, Walterová D (2005). Silybin and silymarin - new effects and applications. Biomed Pap Med Fac Univ Palacky Olomouc Czech Repub.

[16] Juan YS, Levin RM, Chuang SM, Hydery T, Li S, Kogan B, Schuler C, Huang CH, Mannikarottu A (2008). The beneficial effect of coenzyme Q10 and lipoic acid on obstructive bladder dysfunction in the rabbit. J Urol.

[17] Saito M, Miyagawa I (2001). Bladder dysfunction after acute urinary retention in rats. J Urol.

[18] Sehirli O, Sener E, Cetinel S, Yüksel M, Gedik N, Sener G (2008). Alpha-lipoic acid protects against renal ischaemia-reperfusion injury in rats. Clin Exp Pharmacol Physiol.

[19] Hacioglu A, Algin C, Pasaoglu O, Pasaoglu E, Kanbak G (2005). Protective effect of leptin against ischemia-reperfusion injury in the rat small intestine. BMC Gastroenterol.

[20] Erkasap N, Uzuner K, Serteser M, Köken T, Aydin Y (2003). Gastroprotective effect of leptin on gastric mucosal injury induced by ischemia-reperfusion is related to gastric histamine content in rats. Peptides.

[21] Colletti LM, Remick DG, Burtch GD, Kunkel SL, Strieter RM, Campbell DA (1990). Role of tumor necrosis factor-alpha in the pathophysiologic alterations after hepatic ischemia/reperfusion injury in the rat. J Clin Invest.

[22] Kinaci MK, Erkasap N, Kucuk A, Koken T, Tosun M (2012). Effects of quercetin on apoptosis, NF-κB and NOS gene expression in renal ischemia/reperfusion injury. Exp Ther Med.

[23] López-Neblina F, Paez AJ, Toledo AH, Toledo-Pereyra LH (1994). Role of nitric oxide in ischemia/reperfusion of the rat kidney. Circ Shock.

[24] Wan LL, Xia J, Ye D, Liu J, Chen J, Wang G (2009). Effects of quercetin on gene and protein expression on NOX and NOS after myocardial ischemia and reperfusion in rabbit. Cardiovasc Ther.

[25] Martínez-Flórez S, Gutiérrez-Fernández B, Sánchez-Campos S, González-Gallego J, Tuñón MJ (2005). Quercetin attenuates nitric oxide production and nuclear factor kappa B activation in inter-leukin-1 beta activated rat hepatocytes. J Nutr.

[26] Chen H, Xing B, Liu X, Zhan B, Zhou J, Zhu H, Chen Z (2008). Ozone oxidative preconditioning protects the rat kidney from reperfusion injury: the role of nitric oxide. J Surg Res.

[27] Bae EH, Lee KS, Lee J, Ma SK, Kim NH, Choi KC, Frøkiaer J, Nielsen S, Kim SY, Kim SZ, Kim SH, Kim SW (2008). Effects of alpha-lipoic acid on ischemia-reperfusion-induced renal dysfunction in rats. Am J Physiol Renal Physiol.

